# Evolution des paramètres biochimiques et hématologiques chez les personnes vivant avec le VIH/SIDA sous traitement antirétroviral au Centre Médical du Camp General Aboubacar Sangoule Lamizana

**DOI:** 10.11604/pamj.2018.29.159.13659

**Published:** 2018-03-19

**Authors:** Raoul Karfo, Elie Kabré, Laetitia Coulibaly, Georges Diatto, Jean Sakandé, Lassana Sangaré

**Affiliations:** 1 Clinique du Laboratoire de Biologie du Centre Médical du Camp Général Aboubacar Sangoulé Lamizana,Ouagadougou, Burkina Faso; 2 Unité de Formation et de Recherche en Sciences de la Santé, Université de Ouagadougou,Ouagadougou, Burkina Faso; 3 Laboratoire de Biochimie du Centre Hospitalier Universitaire Yalgado Ouedraogo, Ouagadougou, Burkina Faso; 4 Université Saint Thomas d’Aquin (USTA),Ouagadougou, Burkina Faso; 5 Clinique de Dermatologie du Centre Médical du Camp Général Aboubacar Sangoule Lamizana, Ouagadougou, Burkina Faso

**Keywords:** Traitement antirétroviral, VIH / Sida, lymphocytes TCD4, suivi biologique, Antiretroviral therapy, HIV/AIDS, TCD4 lymphocytes, biological monitoring

## Abstract

**Introduction:**

l'objectif de ce travail était d'évaluer l'impact du traitement sur les paramètres biochimiques et hématologiques des patients VIH positifs suivis au Centre Médical du Camp Général Aboubacar Sangoulé Lamizana (CMCGASL) au Burkina Faso.

**Méthodes:**

il s'agissait d'une étude rétrospective réalisée sur la période de Janvier 2010 à septembre 2015. Seuls les patients VIH positifs sous TARV ayant réalisé un bilan biologique à l'une des périodes suivantes : à l'initiation du traitement(M0), au sixième mois(M6) et au douzième mois(M12), ont été pris en compte.

**Résultats:**

le sex-ratio des patients était de 0,88 et la tranche d'âge des 45-55 ans était la plus importante. Les trithérapies incluant 2INTI + 1INNTI (74,5%), 2INTI+1 IP (14,9%) étaient les plus prescrites. La charge virale était peu demandée. Le taux des patients réguliers aux bilans biologiques avait connu une régression statistiquement significative entre M0 (70%), et M6 (13%) (p<0,05). Une augmentation significative de la valeur moyenne des lymphocytes TCD4 allant de 79,22 à M0 à 227,95 cellules/mm3 à M12 (p<0,05) était noté chez les patients sévèrement immunodéprimés. Ascension significative du taux d'hémoglobine moyen passant de 10,5 g/dl à M0 à 12,53 g/dl à M12 (p<0,05) chez les patients qui étaient anémiés. L'analyse des paramètres biochimiques n'a pu être réalisée à cause d'une insuffisance de données.

**Conclusion:**

l'ascension du taux de lymphocytes TCD4 et du taux d'hémoglobine oriente sur l'efficacité du TARV et la tolérance au traitement. Il importe de rendre accessible la charge virale et d'améliorer le suivi biologique.

## Abstract (en)

**Introduction:**

This study aimed to evaluate the impact of treatment on biochemical and hematological parameters in HIV-positive patients followed up at the Aboubacar Sangoulé Lamizana General Camp Medical Center in Burkina Faso.

**Methods:**

We conducted a retrospective study over the period January 2010-September 2015. The study only included HIV-positive patients treated with ART having undergone laboratory tests at treatment initiation (M0), at sixth month (M6), and at twelfth month (M12).

**Results:**

Sex-ratio was 0.88 and the most affected age group was 45-55 years. The most prescribed treatments were triple combination therapies based on 2INTI + 1INNTI (74,5%), 2INTI+1 IP (14,9%). Viral load test was little requested. The rate of patients with normal laboratory tests showed a statistically significant regression between M0 (70%), and M6 (13%) (p<0.05). Severely immunocompromised patients showed a significant increase in average TCD4 lymphocytes value, ranging from 79,22 at M0 to 227.95 cells/mm^3^ at M12 (p<0.05). Anaemic patients showed a significant increase in the rate of mean haemoglobin levels from 10.5 g/dl at M0 to 12.53 g/dl at M12 (p<0.05). The analysis of biochemical parameters could not be performed because of a lack of data.

**Conclusion:**

The increase in the rate of TCD4 lymphocytes and in the rate of hemoglobin levels gives an indication of ART effectiveness and of patient's tolerance to treatment. Viral load test accessibility and an improvement in laboratory testing monitoring are essential.

## Introduction

L´extraordinaire progression du traitement antirétroviral depuis 2010 dans un grand nombre de pays parmi les plus touchés dans le monde a permis de réduire le nombre de décès dus au sida de 1,5 million en 2010 (1,3 million-1,7 million) à 1,1 million (940 000-1,3 million) en 2015 [1]. La couverture mondiale du traitement antirétroviral atteignait 46 % (43-50 %) fin 2015. Les gains ont été les plus importants dans la région la plus touchée du monde, à savoir l´Afrique australe et orientale, où la couverture est passée de 24 % (22-25 %) en 2010 à 54 % (50-58 %) en 2015, atteignant un total de 10,3 millions de personnes [1]. Le rapport ONUSIDA sur l'épidémie mondiale du Sida 2015 indique que la prévalence de l'infection à VIH dans la population adulte du Burkina Faso est estimée à 0,90% en fin 2014, contre 0,92% en fin 2013 [2]. La prévalence de l'infection à VIH dans la population adulte en Guinée, au Mali, en Côte d'ivoire, au Benin, Niger, Ghana et au Togo étaient respectivement de 1,6%, 1,1%, 3%, 1%, 0,5%, 1,7%, 2,3% en 2014. Selon la même source, le nombre de personnes vivant avec le VIH (PVVIH) est estimé à 110 000 dont 95 000 adultes et 13 000 enfants de moins de 15 ans. Parmi ces adultes, 57 000 sont des femmes. Au titre de la prise en charge, on note que le nombre de PVVIH inscrites dans la file active a passé de 76 342 en 2014 à 77 648 PVVIH dont 51 946 femmes en 2015. Quant aux PVVIH sous traitement, elles sont au nombre de 52 248 en 2015 contre 46 623 en fin 2014. Le nombre de décès a connu une baisse importante passant de 991 en 2014 à 634 en fin 2015 soit près du tiers [2]. Le bilan biologique constitue le témoin en temps réel d'une part de l'efficacité et la tolérance du traitement et d'autre part de l'évolution de l'infection. Au Burkina Faso, les patients sous traitement ARV sont confrontés d'une part à un problème de suivi biologique et d'autre part à une rupture fréquente de réactifs. Au regard de toutes ces difficultés dans la prise en charge des PVVIH, nous nous sommes proposés d´évaluer l'impact du traitement sur les paramètres biochimiques et hématologiques des patients VIH positifs suivis Centre Médical du Camp Général Aboubacar Sangoulé Lamizana au Burkina.

## Méthodes

Il s'agissait d'une étude rétrospective réalisée sur la période de Janvier 2010 à septembre 2015 dans les cliniques de dermatologie et des laboratoires du Centre Médical du Camp Aboubacar Sangoulé Lamizana (CMCGASL).

**Population d'étude:** Elle est constituée des Patients VIH positifs adultes sous traitement antirétroviral, initiés et suivis au service de dermatologie du CMCGACL. Le dépistage du VIH avait été effectué conformément à une procédure standard recommandée par les directives nationales sur le dépistage du VIH qui utilisaient deux tests de diagnostic rapide (TDR) dans un algorithme séquentiel. Les échantillons avaient été testés en utilisant un premier TDR (Determine Alere ® HIV1/2). Les échantillons qui s'étaient révélés réactifs avec ce premier TDR ont été évalués par un second test immuno-chromatographique (SD Bioline® HIV1/2 3.0) qui avait permis de préciser le type de VIH (VIH1, VIH2, VIH1 et 2). Seuls les patients VIH positifs sous TARV ayant réalisé un bilan biologique à l'une des périodes suivantes: à l'initiation du traitement(M0), au sixième mois(M6) et au douzième mois(M12), ont été pris en compte. Recueil et analyse des données : Les renseignements (sociodémographiques, les paramètres biologiques...) obtenus ont été recueillis sur une fiche d'enquête. La saisie des données ainsi que le traitement de l'information ont été effectuée sur le logiciel Excel et IBM SPSS Statistics 20. La valeur de p< 0,05 a été considérée comme statistiquement significative. Considérations éthiques : Une base de données anonyme a été constituée à partir des dossiers médicaux et sociaux des patients VIH+ suivis au service de dermatologie du CMCGASL par le CMLS/Défense. Aucune information ne permettait d´identifier les patients inclus dans cette étude. La base de données reste une propriété du Service de dermatologie du CMCGASL et du Comité Ministériel de Lutte contre le VIH/SIDA et les IST (CMLS/Défense). L´étude a été autorisée par le Coordonnateur du CMLS/Défense.

## Résultats

**Caractères sociaux démographiques de la population de l'étude:** Nous avons inclus 94 patients, avec un sex-ratio de 0,88 (Tableau 1). Dans cette étude, l'âge moyen était de 44 ans avec des extrêmes de 16 et de 81 ans. La classe d'âge comprise entre 45-54 ans a été la plus représentative avec (39,4 %) et des taux également élevés, pour les 25-34 ans et les 35-44 ans. Ces trois classes d´âge représentaient 77,64%. La majorité des patients étaient mariés (52,1%). La plus part des patients provenaient de la ville de Ouagadougou avec (63,8%) des cas. La grande majorité des patients était Burkinabè soit 96,8%. La majorité des patients avaient entre 0 et 3 enfants, 11,7% n'avaient aucun enfant et 25,5% des patients étaient des militaires (Tableau 1).

**Tableau 1:** Caractéristiques sociodémographiques de la population d’étude

	Fréquences (n=94)	Pourcentage (%)
**Sexe**		
Féminin	50	53,2
Masculin	44	46,8
**Age (ans)**		
15-24	4	4,3
25-34	18	19,1
35-44	18	19,1
45-54	37	39,4
≥55	15	16
NR	2	2,1
**Statut Social**		
Militaire	24	25,5
Civil	64	68,1
NR	6	6,4
**Statut Matrimonial**		
Marié	49	52,1
Divorcé	2	2,1
Célibataire	35	37,2
Veuf (veuve)	6	6,4
NR	2	2,1
**Lieu de résidence**		
Ouagadougou	60	63,8
Autre	12	12,8
NR	22	23,4
**Nationalité**		
Burkinabé	91	96,8
Autre	1	1,1
NR	2	2,1
**Nombre d’enfants**		
0-3	63	67
4-6	22	23,5
7-9	2	2,1
≥10	2	2,1
NR	5	5,3

NR: Non Renseigné

**Type de VIH et traitement:** Le VIH de type 1 représentait la majorité de l'infection avec 80,9% des cas. Le schéma thérapeutique associant 2 INTI et 1 INNTI était le plus utilisé (74,5%) (Tableau 2).

**Tableau 2:** Type de VIH et traitement

Variable		Fréquences (n = 94)	Pourcentage (%)
**Type de VIH**	VIH-1	76	80,9
	VIH-2	11	11,7
	VIH-1,2	7	7,4
**Schéma thérapeutique au début du traitement**	2INTI+2INNTI	70	74,5
	2INTI+1IP	14	14,9
	3INTI	5	5,3
	1INTI+1INNTI+1IP	1	1,1
	NR	4	4,3

**Paramètres biochimiques et hématologiques:** Le taux des patients réguliers aux bilans biologiques avait connu une régression statistiquement significative entre M0 (77,5%), M6(16,71) (p<0,05) (Tableau 3). En ce qui concerne les charges virales, 6 demandes pour 5 patients avaient été enregistrées: deux patients en 2010, un en 2011, deux en 2015. En 2010 on avait noté deux résultats indétectables. Le seul patient pour lequel la demande avait été faite à M3 puis à M6 a montré une diminution de la charge virale en 2015. A l'inclusion, 45,6% des patients était à un niveau d'immunodépression sévère (LT CD4 <200 cellules/mm³). Après douze mois de traitement ARV on observait une régression statistiquement significative : (45,6 %) à M0; (35,5%) à M6 et (22,9) à M12 (Tableau 4) avec une moyenne de lymphocytes TCD4 allant de 79,22 à M0 à 227,95 à M12. (p<0,05). Le taux des patients ayant une anémie avait connu une régression statistiquement significative: (47,1%) à M0, (36%) à M6, (16,6%) à M12 (Tableau 5) avec un taux d'hémoglobine moyen passant de 10,5 g/dl à M0 à 12,53 g/dl à M12. (p<0,05). Le taux de patients ayant une thrombopénie, n'avait pas connu de changement statistiquement significatif (p>0,05) (Tableau 6).

**Tableau 3:** Répartition des patients selon leur régularité des bilans à MO, M6, M12

Paramètres	MO		M6		M12	
	**Effectif**	**Non réalisé**	**Effectif**	**Non réalisé**	**Effectif**	**Non réalisé**
Lymphocytes TCD4	90	4	31	63	35	59
Hémoglobine	87	7	25	69	18	76
Plaquettes	79	15	24	70	16	78
Créatinine	74	20	10	84	14	80
Amylase	44	50	6	88	5	80
ALAT	79	15	8	86	14	90
Glycémie	57	37	6	88	4	98
Pourcentage (%)	77,5	22,50	16,71	83,29	16,10	83,90

**Tableau 4:** Répartition des patients selon l'évolution du taux de lymphocytes TCD4

	M0	M6	M12
Lymphocytes TCD4 (cellules/mm3)	**Effectif (%)**	**Effectif (%)**	**Effectif (%)**
< 200	41 (45,6)	11 (35,5)	8 (22,9)
200-499	39 (43,3)	15 (48,4)	22 (62,9)
> 500	10 (11,1)	5 (16,1)	5 (14,3)
Total réalisé	90 (100)	31 (100)	35 (100)
Non réalisé	4	69	76
Total	94	94	94

**Tableau 5:** Répartition des patients selon l'évolution du taux d'hémoglobine

	M0	M6	M12
**Hémoglobine (g/dl)**	**Effectif (%)**	**Effectif (%)**	**Effectif (%)**
< 12	41 (47,1)	9 (36)	3 (16,7)
≥ 12	46 (52,9)	16 (64)	15 (83,3)
Total réalisé	87 (100)	25 (100)	18 (100)
Non réalisé	7	69	76
Total	94	94	94

**Tableau 6:** Répartition des patients selon l'évolution du taux de plaquettes

Taux de plaquettes (G/L)	M0	M6	M12
**Plaquettes**	**Effectif (%)**	**Effectif (%)**	**Effectif (%)**
< 150	10 (12,5)	4 (16)	4 (25)
≥ 150	69 (87,5)	20 (84)	12 (75)
Total réalisé	79 (100)	24 (100)	16 (100)
Non réalisé	15	70	78
Total	94	94	94

## Discussion

**Caractères sociaux démographiques de la population de l'étude:** Notre échantillon de 94 patients était constitué à 25,5% de militaires et 68,1% de civils (Tableau 1). Ceci pourrait s'expliquer premièrement par le fait que notre étude s'est déroulée dans une clinique militaire qui accueillait non seulement les militaires mais aussi les civils. Deuxièmement les militaires par soucis de confidentialité préféraient se faire suivre en ville par des structures ou des associations civiles. Les forces armées sont très exposées aux maladies sexuellement transmissibles, dont le VIH pour plusieurs raisons. Comme les soldats passent de longues périodes dans des conditions de stress intense, loin de leurs familles et de leurs foyers, ils risquent davantage que le reste de la population d'avoir des comportements à haut risque, notamment en payant pour avoir des rapports sexuels. Au Burkina Faso, le comité ministériel de lutte contre le VIH/SIDA et les IST section défense (CMLS/défense) avec l'appui du Secrétariat permanent du Conseil national de lutte contre le SIDA et les IST (SP CNLS/IST) et la coopération américaine, a mis l'accent sur la prévention, le dépistage du VIH chez les militaires et leurs familles et le renforcement des capacités des laboratoires pour l'atteinte des objectifs de l'ONUSIDA (90.90.90). Il y avait une prédominance féminine (53,2%) avec un sex-ratio de 0,88 (Tableau 1). La classe d'âge comprise entre 25 et 54 ans était majoritaire (77,64%) avec des extrêmes de 16 et 81 ans. Ces résultats corroborent les données des études réalisées dans d'autres pays. La prédominance féminine a été retrouvée dans l'étude menée par Edith (Mali) [**3**] qui a reporté un sex- ratio de 0,37 en faveur des femmes et comme classe majoritaire celle des 22-44 ans. Les résultats de Bréma [4] au Mali ont reporté comme tranche d'âge majoritaire celle des 25-44 ans. Les patients de la tranche 25-45 ans constituaient 70,7% de l'échantillon et 62,2% de femmes dans l'étude de LOZES et al [5]. Mouhari-Touré et al ont trouvé dans leur étude au Togo une prédominance féminine à 68,6% [6].

Une autre étude menée au Togo montrait la même tranche d'âge la plus touchée par l'infection, avec un pourcentage de 83,7% [5, 7]. Des extrêmes de 18 et 70 ans avec une prédominance féminine a 61,41% avec un sex-ratio (H/F) de 0,62 ont été noté dans l'étude de CISSE et al [8]. La prédominance féminine (68,2%) a été rapportée dans l'étude d'ELIRA Dokekias A et al à Brazzaville [8, 9]. Dans l'étude de NKoumou et al, 62,6% des malades ont un âge compris entre 20 et 50 ans avec une prédominance des patients de sexe féminin [10]. Cette tranche d'âge correspond à celle d'une activité sexuelle maximale exposant aux risques de transmission des infections sexuellement transmissibles. La prédominance du mode de transmission hétérosexuelle dans les régions tropicales, particulièrement en Afrique subsaharienne, peut expliquer la prévalence de la maladie dans cette tranche d'âge [5, 11]. Les mariés représentaient 52,1% de notre échantillon (Tableau 1). Ils représentaient 68,3% dans l'étude de Edith au Mali [3]. Cela pose un problème inquiétant celui du foyer conjugal comme canal de propagation du virus dans les communautés. Cependant, une étude au Gabon a montré une prédominance des célibataires, veufs ou divorces à 62% [8]. Dans la population générale au Gabon, les veufs et les divorcés sont plus infectés que les mariés et les célibataires [10]. Le fait que notre étude se soit déroulée à Ouagadougou a sans doute influencé le fait que 63,8% des patients résidait à Ouagadougou (Tableau 1). Sans grande surprise, la population étudiée est à 96,8% burkinabè étant donné que l'étude s'est déroulée sur le territoire burkinabè. Une étude similaire portant sur une population gabonaise a montré que 83,6% des patients résidaient à Libreville [10].

**Type de VIH et traitement:** Le VIH de type 1 était le plus largement rencontré avec une fréquence de 80,9% des cas (Tableau 2). Un résultat encore plus accentué que le nôtre a été rapporté par Edith au Mali (95,1%) des cas [**3**]. L'étude de CISSE et al a enregistré une large prédominance du VIH de type 1 à 97,07% [8]. Dans L'étude de LOZES, 95,1% des PV VIH de leur échantillon ont été infectées par le VIH 1. Une étude togolaise incluant plus de cinq mille PV VIH à l'initiation du traitement ARV, comptait 97,5% de personnes infectées par le VIH1 [5, 6]. Cette prédominance rapportée par d'autres auteurs s'expliquerait par le fait que le VIH1 est plus répandu, plus virulent et plus transmissible que le VIH 2 [8, 12]. Le schéma thérapeutique associant deux inhibiteurs nucléosidiques de la transcriptase inverse et un inhibiteur non nucléosidique de la transcriptase inverse était le plus utilisé avec 74,5% des cas (Tableau 2). L'étude d'Edith [3] au Mali a reporté 95,1%. Parmi les 436 patients sous ARVs de l'étude de NKoumou et al, 90,1% ont bénéficiés d'une trithérapie (72,9% par 2INTIs+1NNTI, et 17,2% par 2INTIs+1IP) [10].

**Paramètres biochimiques et hématologiques:** Le taux des patients réguliers aux bilans biologiques a connu une régression statistiquement significative entre M0 (77,5%), M6 (16,71%). (p<0,05) (Tableau 3). Cette même constatation a été faite par Bréma [2] au Mali. Cet état de fait est peut-être dû d'une part à une méconnaissance de l'importance du bilan biologique et d'autre part à un manque de moyen financier des patients pour venir au centre. Les lymphocytes T CD4 étaient inférieurs à 200/ mm^3^ dans 45,6% des cas (Tableau 4). Cette situation pourrait s'expliquer par le fait que beaucoup de nos patients étaient vus au stade avancé de l'infection à VIH. Ce déficit immunitaire marqué a été observé par Bréma [4] au Mali qui a rapporté un taux de 51,8%. Après douze mois de traitement ARV on a observé une régression statistiquement significative : (45,6 %) à M0; (35,5%) à M6 et (22,9%) à M12 (Tableau 4) avec une moyenne de lymphocytes TCD4 allant de 79,22 à M0 à 227,95 à M12. (p<0,05). Les résultats obtenus par Bréma [4] au Mali, signifiant un gain considérable de Lymphocytes T CD4 au cours du traitement ARV. L'étude de Ginette CMK et al a constaté une augmentation du taux moyen de toutes les variables étudiées avec une différence significative [13]. Van Dijk et collaborateurs, ont observé également dans leur série une augmentation du taux moyen des CD4 à M6 de 13% par rapport au taux moyen à l'initiation (respectivement 16,3% à M0 et 29,3% à M6) [14]. Reddi et collaborateurs, dans leur étude, observent une augmentation du taux moyen de CD4 de 7,8% à M0 à 18% à M6 [15]. Le nombre moyen de lymphocytes CD4 à l'initiation du traitement des patients de l'étude de Mouhari- Touré et al. était de 134 cellules/ μl contre 137 cellules/ μl dans l'étude de LOZES [6]. Le gain moyen en lymphocytes CD4 des patients de ces études était de plus de 100% entre l'initiation des ARV et les douze mois suivants [5]. L'évaluation de l'efficacité du traitement montre une amélioration des paramètres cliniques. L'un des objectifs du traitement est de restaurer l'immunité. Le suivi biologique met en évidence une augmentation significative du taux de lymphocytes CD4/mm^3^ dès le 6e mois après l'instauration du traitement antirétroviral [10, 13]. La charge virale a très peu été réalisée dans notre étude 6 réalisations pour 5 patients.

Les difficultés liés à la réalisation de ces examens ont été rapportés dans l'étude CISSE et al [8]. L'anémie a été observée dans (47,1%) des cas, à l'inclusion (Tableau 5). Ce taux d'anémie dans l'étude de Bréma au Mali est de 65,9% des cas [4]. Au terme de notre étude, le taux des patients ayant une anémie avait connu une régression statistiquement significative: (47,1%) à M0 à (36%) à M6 à (16,7%) à M12 avec un taux d'hémoglobine moyen passant de 10,5g/dl à M0 à 12,53 g/dl à M12. Par contre Bréma [4] au Mali a notifié un taux d'hémoglobine sans évolution significative. Les plaquettes étaient normales dans 87,5% des cas (Tableau 6). Une thrombopénie a été observée dans (12,5%) des cas. Ces résultats sont proches de ceux d'Edith qui a trouvé (82,9%) de plaquettes normales [3]. Le taux de patients ayant une thrombopénie, n'a pas connu de changement statistiquement significatif. Nos résultats sont proches de ceux de Bréma [4] qui n'a également pas noté d'évolution significative du taux des patients ayant une thrombopénie. L'analyse des résultats biochimiques (l'élévation de l'ALAT, l'Amylase, la créatininémie et la glycémie) parmi ces patients n'a pu être réalisée soit du fait d'une insuffisance de données ou du fait d'une absence de paire valide. En effet ces bilans avaient été réalisés par très peu de patients, et dans les cas où cela avait été réalisé, presqu'aucun contrôle à M6 ou M12 pour ces patients pour nous permettre de suivre leur évolution dans le temps. Les anomalies biologiques sont non seulement dues à l´action directe du VIH lui-même, mais aussi au traitement. Quel que soit le problème rencontré, une modification ou un arrêt des ARV peut être la solution. Des examens sanguins réguliers permettent également d´identifier une toxicité hématologique, hépatique et/ou rénale du traitement. Le taux de CD4 est un indicateur objectif de réponse au traitement antirétroviral. L´augmentation des lymphocytes CD4 peut être lente si la valeur de départ est basse. La mesure de la charge virale lorsqu'elle est réalisable, devra de préférence être couplée avec le dosage des CD4 pour un meilleur suivi.

## Conclusion

La réponse thérapeutique était globalement bonne au terme de cette étude, on a montré une ascension significative du taux d'hémoglobine et de lymphocyte TCD4. Le nombre de personnes régulières au bilan biologique a diminué au cours du traitement. Il faut noter la nécessité de rendre accessible la charge virale et d'améliorer la régularité des bilans biologiques. Cette étude a eu un impact positif sur la stratégie de prise en charge des patients VIH+ suivis au CMCGASL. Le CMLS/Défense, le service de dermatologie et le laboratoire du CMCGASL, ont pris de mesures pour rendre le suivi biologique plus régulier, avec la prise en compte de la charge virale. Des réflexions sont menées au niveau national pour une meilleure accessibilité des patients aux examens biologiques.

### Etat des connaissances actuelles sur le sujet

- Au Burkina Faso, malgré la fréquence du VIH, les données sur l'évolution des facteurs biologiques sont rares et anciennes.

### Contribution de notre étude à la connaissance

- Ce travail fournit des données actualisées sur l'évolution des paramètres biologiques à Ouagadougou (Burkina Faso). Les chiffres retrouvés dans notre étude démontrent l'intérêt d'une étude au niveau national. Cette étude démontre la nécessité d'améliorer la prise en charge biologique de ces patients.

## Conflits d’intérêts

Les auteurs ne déclarent aucun conflit d'intérêts.

## Remerciements

Nous tenons à remercier le personnel des Cliniques des Laboratoires et de Dermatologie du Centre Médical du Camp Général Aboubacar Sangoulé Lamizana au Burkina Faso.
